# Diethyl 7,7′-di­chloro-4-oxo-4*H*-[1,4′-bi­quinoline]-3,3′-di­carboxyl­ate

**DOI:** 10.1107/S1600536814010320

**Published:** 2014-05-10

**Authors:** Yoshinobu Ishikawa, Yasuhiro Sugisawa

**Affiliations:** a School of Pharmaceutical Sciences, University of Shizuoka, 52-1 Yada, Suruga-ku, Shizuoka 422-8526, Japan

## Abstract

In the title compound, C_24_H_18_Cl_2_N_2_O_5_, the quinoline and quinolinone moieties are nearly perpendicular to each other, forming a dihedral angle of 82.36 (3)°. In the crystal, mol­ecules form a halogen bond between a Cl atom of a quinolinone moiety and the N atom of the quinoline moiety of the inversion equivalent [Cl⋯N = 3.106 (3) Å]. The mol­ecules also form two kinds of C—H⋯O hydrogen-bonded centrosymmetric inversion dimers, making chains along the *c-*axis direction which are further inter­linked by the halogen bonds into layers parallel to the *bc* plane.

## Related literature   

For background to this study, see: Ishikawa & Fujii (2011 ▶). For a related structure, see: Ishikawa & Yoshida (2014 ▶).
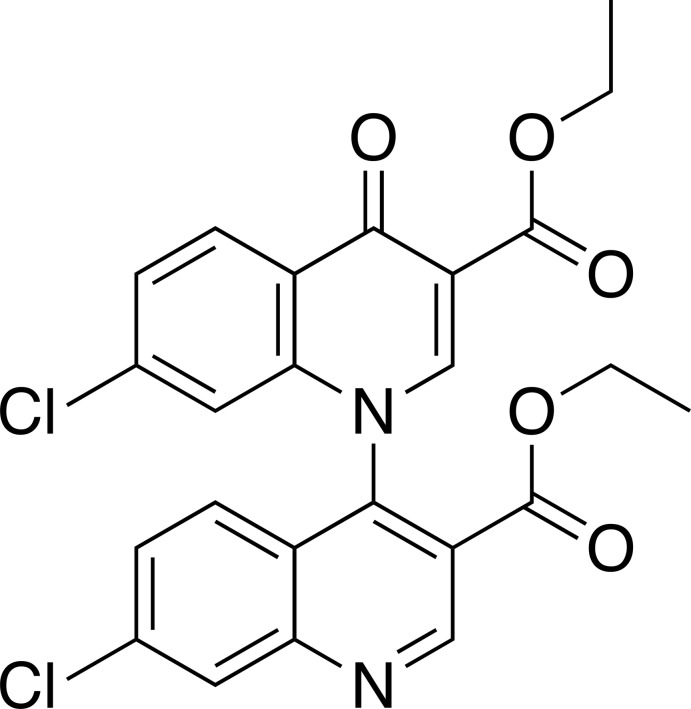


## Experimental   

### 

#### Crystal data  

C_24_H_18_Cl_2_N_2_O_5_
*M* *_r_* = 485.32
Triclinic, 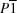

*a* = 7.631 (4) Å
*b* = 12.416 (5) Å
*c* = 12.461 (5) Å
α = 107.99 (3)°
β = 101.08 (3)°
γ = 94.77 (3)°
*V* = 1089.0 (8) Å^3^
*Z* = 2
Mo *K*α radiation
μ = 0.34 mm^−1^
*T* = 100 K
0.35 × 0.25 × 0.10 mm

#### Data collection  

Rigaku AFC-7R diffractometer
6003 measured reflections
4994 independent reflections
4229 reflections with *F* ^2^ > 2σ(*F* ^2^)
*R*_int_ = 0.059
3 standard reflections every 150 reflections intensity decay: 1.7%

#### Refinement  

*R*[*F* ^2^ > 2σ(*F* ^2^)] = 0.060
*wR*(*F* ^2^) = 0.186
*S* = 1.10
4994 reflections
300 parameters
H-atom parameters constrained
Δρ_max_ = 0.66 e Å^−3^
Δρ_min_ = −0.95 e Å^−3^

### 

Data collection: *WinAFC Diffractometer Control Software* (Rigaku, 1999 ▶); cell refinement: *WinAFC Diffractometer Control Software*; data reduction: *WinAFC Diffractometer Control Software*; program(s) used to solve structure: *SIR2008* (Burla *et al.*, 2007 ▶); program(s) used to refine structure: *SHELXL97* (Sheldrick, 2008 ▶); molecular graphics: *CrystalStructure* (Rigaku, 2010 ▶); software used to prepare material for publication: *CrystalStructure*.

## Supplementary Material

Crystal structure: contains datablock(s) General, I. DOI: 10.1107/S1600536814010320/ld2125sup1.cif

Structure factors: contains datablock(s) I. DOI: 10.1107/S1600536814010320/ld2125Isup2.hkl

Supporting information file. DOI: 10.1107/S1600536814010320/ld2125Isup3.cml

CCDC reference: 1001457

Additional supporting information:  crystallographic information; 3D view; checkCIF report

## Figures and Tables

**Table 1:** Hydrogen-bond geometry (Å, °)

*D*—H⋯*A*	*D*—H	H⋯*A*	*D*⋯*A*	*D*—H⋯*A*
C16—H11⋯O1^i^	0.95	2.35	3.231 (3)	155 (1)
C13—H10⋯O2^ii^	0.95	2.38	3.301 (4)	162 (1)

Symmetry codes: (i) 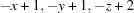; (ii) 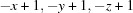.

**Fig. 1.**
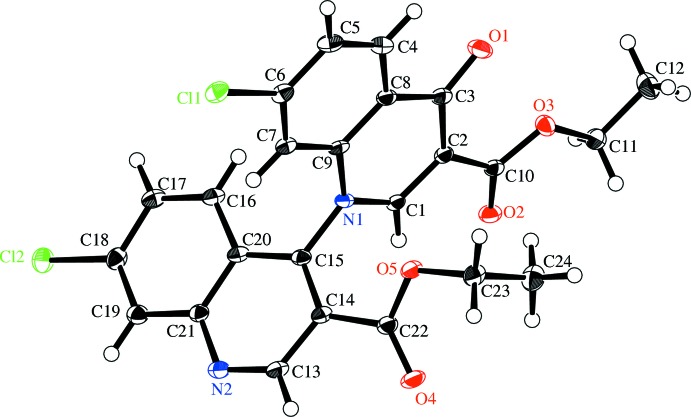
The molecular structure of the title compound with displacement ellipsoids drawn at the 50% probability level. Hydrogen atoms are shown as small spheres of arbitrary radius.

**Fig. 2.**
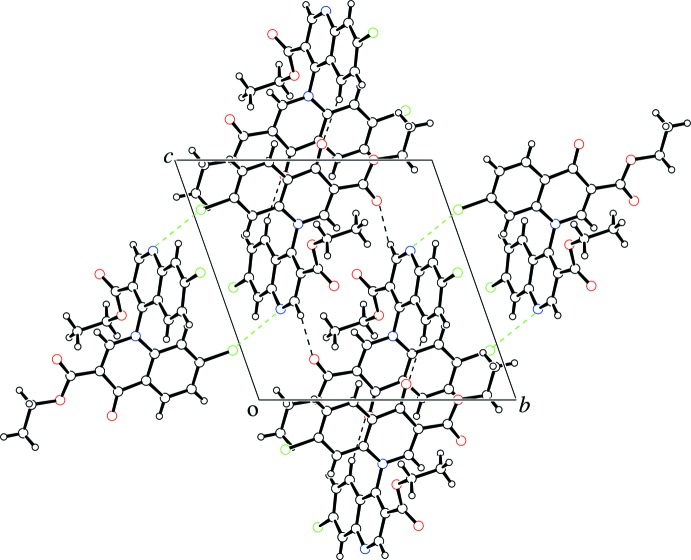
A view of the intermolecular interactions of the title compound, represented as dashed green lines for Cl···N and dashed black lines for C–H···O interactions.

## supplementary crystallographic information

### 1. Comment

4-Quinolones show inhibition not only to Gram negative and Gram positive bacteria, but also to human immunodeficiency virus (HIV). The inhibition to HIV is derived from their chelating ability to metal ions in the active site of metalloenzyme HIV integrase. According to our inhibitor design targeting metalloenzyme influenza virus RNA polymerase (Ishikawa & Fujii, 2011), we tried to synthesize a 4-quinolone derivative bearing a benzenesulfonyl group. The crystallographic analysis revealed that the reaction of ethyl 4-oxo-1,4-dihydroquinoline-3-carboxylate with benzenesulfonyl chloride in the presence of K_2_CO_3_ in *N*,*N*-dimethylformamide (DMF) at 120 °C provided an unexpected 1,4'-biquinoline derivative, diethyl 4-oxo-4*H*-[1,4'-biquinoline]-3,3'-dicarboxylate (Ishikawa & Yoshida, 2014). 1,4'-Biquinoline derivatives might be potential enzyme inhibitors. Thus, we synthesized the title compound, a dichlorinated 1,4'-biquinoline derivative, by the similar method mentioned above, and herein report its crystal structure.

As shown in Fig.1, the C–N bond formation between the quinolinone and quinoline rings is confirmed. The chloroquinolinone and chloroquinoline moieties are nearly perpendicular to each other [dihedral angle = 97.64 (3)°]. In the crystal, the molecules are linked to each other to give dimers through halogen bond between the Cl atoms of the chloroquinolinone moieties and the N atoms of the chloroquinoline moieties of the inversion equivalents^i^ [Cl1···N2 = 3.106 (3) Å, i: –*x* + 1, –*y*, –*z* + 1]. Two systems of C–H···O hydrogen-bonded dimers form chains along the *c*-axis, which are interlinked by the halogen bonds forming layers parallel to the *bc* plane, as shown in Fig.2. On the other hand, clear-cut ring-ring stacking interaction is not found. These findings are in contrast with those in the crystal packing of diethyl 4-oxo-4*H*-[1,4'-biquinoline]-3,3'-dicarboxylate (Ishikawa & Yoshida, 2014).

### 2. Experimental

In a Schlenk tube under nitrogen atmosphere, the mixture of ethyl 7-chloro-4-oxo-1,4-dihydroquinoline-3-carboxylate (5.00 mmol), benzenesulfonyl chloride (5.00 mmol), K_2_CO_3_ (10.0 mmol) in 10 ml of DMF were stirred at 130 °C overnight. After cooling to room temperature ice water was added. The precipitates were collected, and were recrystallized from DMF to give white solids (yield: 18%). ^1^H NMR (400 MHz, DMSO-*d*_6_): *δ* = 0.90 (t, 3H, *J* = 7.0 Hz), 1.23 (t, 3H, *J* = 7.0 Hz), 4.03–4.15 (m, 2H), 4.20 (q, 2H, *J* = 7.0 Hz), 6.99 (d, 1H, *J* = 1.4 Hz), 7.52 (dd, 1H, *J* = 1.4 and 8.3 Hz), 7.74 (br s, 2H), 8.31 (d, 1H, *J* = 8.3 Hz), 8.43 (s, 1H), 8.70 (s, 1H), 9.57 (s, 1H). DART-MS calcd for [C_24_H_18_Cl_2_N_2_O_5_ + H^+^]: 484.059, found 485.099. Single crystals suitable for X-ray diffraction were obtained by slow evapolation of an ethyl acetate solution of the title compound at room temperature.

### 3. Refinement

The C(*sp*^2^)-bound [C–H 0.95 Å, *U*_iso_(H) = 1.2*U*_eq_(C)] and methylene [C–H 0.99 Å, *U*_iso_(H) = 1.2*U*_eq_(C)] hydrogen atoms were placed in geometrical positions and refined using a riding model. A rotating group model was applied to the methyl groups with distance constraint [C–H = 0.98 Å, *U*_iso_(H) = 1.2*U*_eq_(C)].

### Crystal data

**Table:**

C_24_H_18_Cl_2_N_2_O_5_	*Z* = 2
*M**_r_* = 485.32	*F*(000) = 500.00
Triclinic, *P*1	*D*_x_ = 1.480 Mg m^−^^3^
Hall symbol: -P 1	Mo *K*α radiation, λ = 0.71069 Å
*a* = 7.631 (4) Å	Cell parameters from 25 reflections
*b* = 12.416 (5) Å	θ = 15.3–17.4°
*c* = 12.461 (5) Å	µ = 0.34 mm^−^^1^
α = 107.99 (3)°	*T* = 100 K
β = 101.08 (3)°	Plate, colorless
γ = 94.77 (3)°	0.35 × 0.25 × 0.10 mm
*V* = 1089.0 (8) Å^3^	

### Data collection

**Table:**

Rigaku AFC-7R diffractometer	θ_max_ = 27.5°
ω–2θ scans	*h* = −9→9
6003 measured reflections	*k* = −15→16
4994 independent reflections	*l* = −16→9
4229 reflections with *F*^2^ > 2σ(*F*^2^)	3 standard reflections every 150 reflections
*R*_int_ = 0.059	intensity decay: 1.7%

### Refinement

**Table:**

Refinement on *F*^2^	Secondary atom site location: difference Fourier map
*R*[*F*^2^ > 2σ(*F*^2^)] = 0.060	Hydrogen site location: inferred from neighbouring sites
*wR*(*F*^2^) = 0.186	H-atom parameters constrained
*S* = 1.10	*w* = 1/[σ^2^(*F*_o_^2^) + (0.1156*P*)^2^ + 1.0113*P*] where *P* = (*F*_o_^2^ + 2*F*_c_^2^)/3
4994 reflections	(Δ/σ)_max_ < 0.001
300 parameters	Δρ_max_ = 0.66 e Å^−^^3^
0 restraints	Δρ_min_ = −0.95 e Å^−^^3^
Primary atom site location: structure-invariant direct methods	

### Special details

**Table:**

Refinement. Refinement was performed using all reflections. The weighted *R*-factor (*wR*) and goodness of fit (*S*) are based on *F*^2^. *R*-factor (gt) are based on *F*. The threshold expression of *F*^2^ > 2.0 σ(*F*^2^) is used only for calculating *R*-factor (gt).

### Fractional atomic coordinates and isotropic or equivalent isotropic displacement parameters (Å^2^)

**Table:**

	*x*	*y*	*z*	*U*_iso_*/*U*_eq_	
Cl1	0.73301 (8)	0.03469 (5)	0.79333 (5)	0.02074 (18)	
Cl2	−0.29884 (8)	0.05407 (6)	0.46908 (5)	0.02351 (19)	
O1	0.7561 (3)	0.59358 (16)	1.06621 (15)	0.0228 (4)	
O2	0.4484 (3)	0.72976 (15)	0.83028 (15)	0.0197 (4)	
O3	0.6483 (3)	0.77390 (15)	1.00077 (15)	0.0198 (4)	
O4	0.7338 (3)	0.44766 (18)	0.47536 (16)	0.0261 (5)	
O5	0.8082 (3)	0.41909 (16)	0.64744 (15)	0.0182 (4)	
N1	0.5095 (3)	0.39265 (17)	0.73369 (16)	0.0138 (4)	
N2	0.2732 (3)	0.20828 (18)	0.37892 (17)	0.0174 (5)	
C1	0.5012 (3)	0.5061 (2)	0.7706 (2)	0.0141 (5)	
C2	0.5822 (3)	0.5797 (2)	0.8794 (2)	0.0145 (5)	
C3	0.6857 (4)	0.5365 (2)	0.9646 (2)	0.0163 (5)	
C4	0.8031 (4)	0.3633 (3)	0.9917 (2)	0.0198 (5)	
C5	0.8157 (4)	0.2478 (3)	0.9544 (3)	0.0208 (5)	
C6	0.7215 (4)	0.1800 (2)	0.8429 (2)	0.0175 (5)	
C7	0.6192 (3)	0.2257 (2)	0.7685 (2)	0.0157 (5)	
C8	0.6992 (3)	0.4127 (2)	0.9191 (2)	0.0154 (5)	
C9	0.6093 (3)	0.3430 (2)	0.80736 (19)	0.0142 (5)	
C10	0.5507 (4)	0.6999 (2)	0.8995 (2)	0.0152 (5)	
C11	0.6218 (4)	0.8920 (2)	1.0198 (3)	0.0202 (5)	
C12	0.7347 (4)	0.9628 (3)	1.1383 (3)	0.0272 (6)	
C13	0.4320 (4)	0.2725 (3)	0.4112 (2)	0.0178 (5)	
C14	0.5195 (4)	0.3360 (2)	0.5282 (2)	0.0157 (5)	
C15	0.4338 (3)	0.32682 (19)	0.61337 (19)	0.0141 (5)	
C16	0.1675 (4)	0.2425 (3)	0.6666 (2)	0.0179 (5)	
C17	−0.0004 (4)	0.1789 (3)	0.6309 (3)	0.0193 (5)	
C18	−0.0794 (4)	0.1281 (3)	0.5107 (3)	0.0190 (5)	
C19	0.0106 (4)	0.1366 (2)	0.4280 (2)	0.0185 (5)	
C20	0.2636 (4)	0.2570 (2)	0.5836 (2)	0.0158 (5)	
C21	0.1866 (4)	0.2007 (2)	0.4634 (2)	0.0156 (5)	
C22	0.6974 (4)	0.4081 (2)	0.5471 (2)	0.0171 (5)	
C23	0.9888 (4)	0.4845 (3)	0.6723 (3)	0.0203 (5)	
C24	0.9854 (4)	0.6121 (3)	0.7135 (3)	0.0259 (6)	
H1	0.4346	0.5369	0.7175	0.0170*	
H2	0.8658	0.4101	1.0677	0.0238*	
H3	0.8873	0.2151	1.0037	0.0250*	
H4	0.5570	0.1784	0.6926	0.0188*	
H5A	0.4926	0.8991	1.0160	0.0243*	
H6B	0.6599	0.9189	0.9596	0.0243*	
H7A	0.6963	0.9352	1.1971	0.0326*	
H8B	0.7188	1.0433	1.1540	0.0326*	
H9C	0.8623	0.9559	1.1408	0.0326*	
H10	0.4926	0.2769	0.3525	0.0213*	
H11	0.2205	0.2771	0.7470	0.0215*	
H12	−0.0642	0.1687	0.6863	0.0232*	
H13	−0.0441	0.1001	0.3480	0.0222*	
H14A	1.0365	0.4633	0.6013	0.0244*	
H15B	1.0710	0.4644	0.7325	0.0244*	
H16A	1.1094	0.6531	0.7397	0.0310*	
H17B	0.9245	0.6318	0.7779	0.0310*	
H18C	0.9200	0.6340	0.6497	0.0310*	

### Atomic displacement parameters (Å^2^)

**Table:**

	*U*^11^	*U*^22^	*U*^33^	*U*^12^	*U*^13^	*U*^23^
Cl1	0.0250 (4)	0.0193 (3)	0.0227 (4)	0.0070 (3)	0.0079 (3)	0.0114 (3)
Cl2	0.0156 (3)	0.0274 (4)	0.0264 (4)	−0.0013 (3)	0.0043 (3)	0.0089 (3)
O1	0.0318 (11)	0.0220 (10)	0.0129 (9)	0.0056 (8)	0.0014 (7)	0.0052 (7)
O2	0.0249 (9)	0.0203 (9)	0.0161 (9)	0.0049 (7)	0.0034 (7)	0.0094 (7)
O3	0.0249 (10)	0.0147 (9)	0.0170 (9)	0.0023 (7)	−0.0005 (7)	0.0045 (7)
O4	0.0241 (10)	0.0375 (12)	0.0205 (10)	−0.0052 (8)	0.0041 (8)	0.0181 (9)
O5	0.0144 (8)	0.0252 (9)	0.0180 (9)	0.0003 (7)	0.0028 (7)	0.0126 (7)
N1	0.0160 (10)	0.0165 (10)	0.0101 (9)	0.0026 (8)	0.0030 (7)	0.0063 (8)
N2	0.0204 (10)	0.0194 (10)	0.0140 (10)	0.0037 (8)	0.0043 (8)	0.0073 (8)
C1	0.0155 (11)	0.0163 (11)	0.0130 (10)	0.0012 (9)	0.0054 (8)	0.0074 (9)
C2	0.0164 (11)	0.0154 (11)	0.0140 (11)	0.0022 (9)	0.0059 (9)	0.0067 (9)
C3	0.0183 (11)	0.0196 (12)	0.0133 (11)	0.0029 (9)	0.0067 (9)	0.0069 (9)
C4	0.0227 (12)	0.0239 (13)	0.0145 (11)	0.0044 (10)	0.0036 (9)	0.0087 (10)
C5	0.0243 (13)	0.0254 (13)	0.0182 (12)	0.0092 (10)	0.0053 (10)	0.0134 (10)
C6	0.0194 (12)	0.0187 (12)	0.0188 (12)	0.0059 (9)	0.0085 (9)	0.0092 (10)
C7	0.0164 (11)	0.0190 (12)	0.0149 (11)	0.0036 (9)	0.0062 (9)	0.0083 (9)
C8	0.0154 (11)	0.0201 (12)	0.0126 (11)	0.0019 (9)	0.0047 (9)	0.0076 (9)
C9	0.0145 (11)	0.0201 (12)	0.0126 (11)	0.0036 (9)	0.0065 (8)	0.0096 (9)
C10	0.0177 (11)	0.0172 (11)	0.0128 (11)	0.0023 (9)	0.0065 (9)	0.0060 (9)
C11	0.0263 (13)	0.0153 (12)	0.0188 (12)	0.0034 (10)	0.0033 (10)	0.0065 (9)
C12	0.0307 (15)	0.0186 (13)	0.0255 (14)	0.0033 (11)	−0.0024 (11)	0.0032 (11)
C13	0.0185 (12)	0.0234 (12)	0.0157 (11)	0.0038 (9)	0.0064 (9)	0.0108 (10)
C14	0.0170 (11)	0.0180 (11)	0.0153 (11)	0.0039 (9)	0.0040 (9)	0.0094 (9)
C15	0.0166 (11)	0.0151 (11)	0.0126 (11)	0.0044 (9)	0.0024 (8)	0.0074 (9)
C16	0.0194 (12)	0.0224 (12)	0.0156 (11)	0.0042 (9)	0.0049 (9)	0.0106 (10)
C17	0.0191 (12)	0.0249 (13)	0.0190 (12)	0.0058 (10)	0.0085 (9)	0.0114 (10)
C18	0.0134 (11)	0.0220 (12)	0.0233 (13)	0.0011 (9)	0.0032 (9)	0.0108 (10)
C19	0.0182 (12)	0.0211 (12)	0.0170 (12)	0.0028 (9)	0.0023 (9)	0.0085 (10)
C20	0.0174 (11)	0.0193 (12)	0.0143 (11)	0.0052 (9)	0.0043 (9)	0.0098 (9)
C21	0.0162 (11)	0.0167 (11)	0.0154 (11)	0.0023 (9)	0.0016 (9)	0.0086 (9)
C22	0.0177 (11)	0.0199 (12)	0.0157 (11)	0.0019 (9)	0.0055 (9)	0.0078 (9)
C23	0.0128 (11)	0.0272 (13)	0.0233 (13)	0.0010 (9)	0.0032 (9)	0.0126 (11)
C24	0.0223 (13)	0.0239 (14)	0.0322 (15)	0.0027 (10)	0.0006 (11)	0.0141 (12)

### Geometric parameters (Å, º)

**Table:**

Cl1—C6	1.733 (3)	C14—C22	1.498 (4)
Cl2—C18	1.742 (3)	C15—C20	1.416 (4)
O1—C3	1.231 (3)	C16—C17	1.364 (4)
O2—C10	1.216 (4)	C16—C20	1.423 (4)
O3—C10	1.341 (3)	C17—C18	1.418 (4)
O3—C11	1.450 (4)	C18—C19	1.368 (5)
O4—C22	1.209 (4)	C19—C21	1.418 (4)
O5—C22	1.329 (3)	C20—C21	1.422 (3)
O5—C23	1.466 (3)	C23—C24	1.511 (4)
N1—C1	1.351 (4)	C1—H1	0.950
N1—C9	1.400 (4)	C4—H2	0.950
N1—C15	1.443 (3)	C5—H3	0.950
N2—C13	1.311 (4)	C7—H4	0.950
N2—C21	1.368 (4)	C11—H5A	0.990
C1—C2	1.369 (3)	C11—H6B	0.990
C2—C3	1.462 (4)	C12—H7A	0.980
C2—C10	1.483 (4)	C12—H8B	0.980
C3—C8	1.484 (4)	C12—H9C	0.980
C4—C5	1.383 (4)	C13—H10	0.950
C4—C8	1.404 (4)	C16—H11	0.950
C5—C6	1.396 (4)	C17—H12	0.950
C6—C7	1.382 (4)	C19—H13	0.950
C7—C9	1.400 (4)	C23—H14A	0.990
C8—C9	1.396 (3)	C23—H15B	0.990
C11—C12	1.510 (4)	C24—H16A	0.980
C13—C14	1.421 (3)	C24—H17B	0.980
C14—C15	1.377 (4)	C24—H18C	0.980
			
O1···O3	2.744 (3)	N1···H15B^v^	3.5315
O1···C1	3.599 (4)	N2···H17B^iv^	3.4348
O1···C4	2.797 (4)	N2···H18C^iv^	2.6158
O1···C10	3.050 (4)	C1···H10^iv^	3.4894
O2···C1	2.730 (4)	C1···H15B^v^	3.1990
O2···C11	2.608 (3)	C3···H2^xi^	3.5544
O3···C1	3.576 (3)	C3···H11^iii^	3.5186
O3···C3	2.889 (4)	C4···H15B^xi^	3.3403
O4···C13	2.839 (4)	C4···H16A^xi^	3.3578
O4···C23	2.710 (4)	C4···H17B^xi^	3.1853
O4···C24	3.174 (4)	C5···H5A^iii^	3.0056
O5···N1	2.737 (3)	C5···H12^vii^	3.5018
O5···C1	3.129 (4)	C5···H16A^xi^	3.5386
O5···C7	3.552 (4)	C5···H17B^xi^	3.3435
O5···C9	3.042 (4)	C6···H5A^iii^	2.9375
O5···C13	3.577 (4)	C6···H7A^iii^	3.2632
O5···C15	2.888 (4)	C6···H12^vii^	2.7567
N1···C3	2.866 (3)	C7···H7A^iii^	3.1775
N1···C16	2.894 (4)	C7···H12^vii^	2.8588
N1···C22	3.002 (4)	C10···H2^iii^	3.5006
N2···C15	2.806 (3)	C10···H10^iv^	3.1987
C1···C8	2.756 (4)	C10···H16A^v^	3.4673
C1···C14	3.143 (4)	C11···H5A^viii^	2.9378
C1···C20	3.358 (4)	C11···H6B^viii^	3.2975
C1···C22	3.360 (4)	C11···H8B^viii^	3.3685
C2···C9	2.833 (4)	C12···H5A^viii^	3.3011
C4···C7	2.791 (4)	C12···H13^xiii^	2.7669
C5···C9	2.788 (4)	C13···H1^iv^	3.4318
C6···C8	2.780 (4)	C13···H18C^iv^	3.0680
C7···C15	2.839 (4)	C15···H14A^v^	3.5947
C7···C16	3.484 (4)	C16···H7A^iii^	3.2940
C7···C20	3.339 (4)	C16···H14A^v^	3.2712
C9···C14	3.386 (4)	C16···H15B^v^	2.8250
C9···C16	3.417 (4)	C17···H7A^iii^	3.5354
C9···C20	3.277 (4)	C17···H13^ix^	3.5923
C13···C19	3.578 (4)	C17···H15B^v^	3.3327
C13···C20	2.752 (5)	C19···H7A^xii^	3.4912
C14···C21	2.755 (4)	C19···H8B^xii^	3.4869
C16···C19	2.821 (4)	C19···H9C^xii^	3.4902
C17···C21	2.809 (5)	C19···H18C^iv^	3.3135
C18···C20	2.780 (4)	C20···H14A^v^	3.1857
C22···C24	3.102 (4)	C20···H15B^v^	3.3105
Cl1···N2^i^	3.106 (3)	C21···H14A^v^	3.5795
Cl2···Cl2^ii^	3.5616 (17)	C21···H18C^iv^	2.9127
O1···N1^iii^	3.477 (4)	C22···H1^iv^	3.5465
O1···C1^iii^	3.494 (4)	C22···H14A^vi^	3.5804
O1···C2^iii^	3.521 (4)	C23···H1^vii^	3.3137
O1···C3^iii^	3.512 (4)	C23···H2^xi^	3.0336
O1···C8^iii^	3.509 (4)	C23···H11^vii^	3.4964
O1···C9^iii^	3.487 (4)	C24···H2^xi^	2.8503
O1···C16^iii^	3.231 (3)	C24···H3^xi^	3.4109
O2···C4^iii^	3.577 (4)	H1···O4^iv^	2.5687
O2···C13^iv^	3.301 (4)	H1···C13^iv^	3.4318
O2···C24^v^	3.548 (4)	H1···C22^iv^	3.5465
O4···C1^iv^	3.476 (4)	H1···C23^v^	3.3137
O4···C14^iv^	3.443 (4)	H1···H10^iv^	2.7626
O4···C15^iv^	3.558 (4)	H1···H14A^v^	3.0313
O4···C23^vi^	3.276 (4)	H1···H15B^v^	2.9039
O4···C24^vi^	3.436 (4)	H1···H16A^v^	2.9874
O5···C17^vii^	3.395 (4)	H2···O1^xi^	3.5966
N1···O1^iii^	3.477 (4)	H2···O2^iii^	3.4958
N2···Cl1^i^	3.106 (3)	H2···C3^xi^	3.5544
N2···C24^iv^	3.412 (4)	H2···C10^iii^	3.5006
C1···O1^iii^	3.494 (4)	H2···C23^xi^	3.0336
C1···O4^iv^	3.476 (4)	H2···C24^xi^	2.8503
C2···O1^iii^	3.521 (4)	H2···H15B^xi^	2.4244
C2···C3^iii^	3.578 (5)	H2···H16A^xi^	2.7246
C2···C8^iii^	3.589 (4)	H2···H17B^xi^	2.4712
C3···O1^iii^	3.512 (4)	H3···O2^iii^	3.5724
C3···C2^iii^	3.578 (5)	H3···O3^xi^	3.5471
C3···C3^iii^	3.264 (4)	H3···C24^xi^	3.4109
C3···C8^iii^	3.535 (4)	H3···H5A^iii^	3.0498
C4···O2^iii^	3.577 (4)	H3···H8B^x^	3.5569
C4···C10^iii^	3.384 (5)	H3···H9C^xi^	3.3216
C6···C11^iii^	3.587 (5)	H3···H16A^xi^	3.1037
C8···O1^iii^	3.509 (4)	H3···H17B^xi^	2.8161
C8···C2^iii^	3.589 (4)	H4···Cl2^vii^	3.1862
C8···C3^iii^	3.535 (4)	H4···Cl2^ix^	3.1952
C9···O1^iii^	3.487 (4)	H4···H7A^iii^	3.0634
C10···C4^iii^	3.384 (5)	H4···H12^vii^	2.9178
C11···C6^iii^	3.587 (5)	H5A···Cl1^iii^	3.1443
C11···C11^viii^	3.490 (5)	H5A···C5^iii^	3.0056
C13···O2^iv^	3.301 (4)	H5A···C6^iii^	2.9375
C14···O4^iv^	3.443 (4)	H5A···C11^viii^	2.9378
C15···O4^iv^	3.558 (4)	H5A···C12^viii^	3.3011
C16···O1^iii^	3.231 (3)	H5A···H3^iii^	3.0498
C16···C23^v^	3.388 (5)	H5A···H5A^viii^	2.6538
C17···O5^v^	3.395 (4)	H5A···H6B^viii^	2.5893
C18···C18^ix^	3.450 (4)	H5A···H8B^viii^	2.7235
C23···O4^vi^	3.276 (4)	H6B···Cl1^xiv^	2.9705
C23···C16^vii^	3.388 (5)	H6B···C11^viii^	3.2975
C24···O2^vii^	3.548 (4)	H6B···H5A^viii^	2.5893
C24···O4^vi^	3.436 (4)	H6B···H6B^viii^	3.3956
C24···N2^iv^	3.412 (4)	H6B···H8B^viii^	3.1042
Cl1···H3	2.8323	H7A···Cl1^iii^	3.3484
Cl1···H4	2.7746	H7A···Cl2^xiii^	3.2436
Cl2···H12	2.8089	H7A···C6^iii^	3.2632
Cl2···H13	2.8031	H7A···C7^iii^	3.1775
O1···H2	2.4958	H7A···C16^iii^	3.2940
O2···H1	2.3541	H7A···C17^iii^	3.5354
O2···H5A	2.5424	H7A···C19^xiii^	3.4912
O2···H6B	2.5978	H7A···H4^iii^	3.0634
O3···H7A	2.5759	H7A···H11^iii^	3.0133
O3···H8B	3.2380	H7A···H12^iii^	3.4423
O3···H9C	2.5830	H7A···H13^xiii^	2.6772
O4···H10	2.5478	H8B···O2^viii^	3.1553
O4···H14A	2.4861	H8B···C11^viii^	3.3685
O4···H18C	2.6932	H8B···C19^xiii^	3.4869
O5···H1	3.4480	H8B···H3^xiv^	3.5569
O5···H16A	3.2950	H8B···H5A^viii^	2.7235
O5···H17B	2.6098	H8B···H6B^viii^	3.1042
O5···H18C	2.7231	H8B···H13^xiii^	2.5821
N1···H4	2.6190	H9C···Cl1^xi^	3.0218
N1···H11	2.5903	H9C···C19^xiii^	3.4902
N2···H13	2.5685	H9C···H3^xi^	3.3216
C1···H11	3.3147	H9C···H12^iii^	3.2541
C1···H17B	3.4411	H9C···H13^xiii^	2.5698
C2···H17B	3.2151	H10···O2^iv^	2.3840
C3···H1	3.3018	H10···C1^iv^	3.4894
C3···H2	2.6316	H10···C10^iv^	3.1987
C5···H4	3.2843	H10···H1^iv^	2.7626
C6···H2	3.2504	H10···H16A^vi^	3.5807
C7···H3	3.2817	H10···H18C^iv^	3.4202
C7···H11	3.1390	H11···O1^iii^	2.3474
C8···H3	3.2855	H11···O3^iii^	3.3776
C8···H4	3.2917	H11···C3^iii^	3.5186
C9···H1	3.2331	H11···C23^v^	3.4964
C9···H2	3.2661	H11···H7A^iii^	3.0133
C9···H11	2.8974	H11···H14A^v^	3.5728
C10···H1	2.4741	H11···H15B^v^	2.7129
C10···H5A	2.5743	H12···Cl1^v^	2.9460
C10···H6B	2.6022	H12···O5^v^	3.4785
C10···H17B	3.5241	H12···C5^v^	3.5018
C14···H1	3.0701	H12···C6^v^	2.7567
C14···H4	3.2328	H12···C7^v^	2.8588
C15···H1	2.5282	H12···H4^v^	2.9178
C15···H4	2.5115	H12···H7A^iii^	3.4423
C15···H10	3.2423	H12···H9C^iii^	3.2541
C15···H11	2.7043	H12···H13^ix^	3.4222
C16···H4	3.1231	H12···H15B^v^	3.5653
C17···H13	3.2952	H13···Cl1^i^	3.4612
C18···H11	3.2670	H13···C12^xii^	2.7669
C19···H12	3.2857	H13···C17^ix^	3.5923
C20···H1	3.3755	H13···H7A^xii^	2.6772
C20···H4	2.7971	H13···H8B^xii^	2.5821
C20···H12	3.2767	H13···H9C^xii^	2.5698
C20···H13	3.3143	H13···H12^ix^	3.4222
C21···H10	3.1520	H13···H16A^iv^	3.5989
C21···H11	3.3155	H13···H18C^iv^	3.3482
C22···H1	3.3402	H14A···O4^vi^	2.4859
C22···H10	2.5803	H14A···C15^vii^	3.5947
C22···H14A	2.5239	H14A···C16^vii^	3.2712
C22···H15B	3.1771	H14A···C20^vii^	3.1857
C22···H17B	3.3676	H14A···C21^vii^	3.5795
C22···H18C	2.9232	H14A···C22^vi^	3.5804
H2···H3	2.3340	H14A···H1^vii^	3.0313
H4···H11	3.0391	H14A···H11^vii^	3.5728
H5A···H7A	2.3705	H14A···H14A^vi^	2.9112
H5A···H8B	2.3637	H14A···H18C^vi^	3.0792
H5A···H9C	2.8598	H15B···O1^xi^	2.9240
H6B···H7A	2.8598	H15B···N1^vii^	3.5315
H6B···H8B	2.3689	H15B···C1^vii^	3.1990
H6B···H9C	2.3653	H15B···C4^xi^	3.3403
H11···H12	2.3146	H15B···C16^vii^	2.8250
H14A···H16A	2.3955	H15B···C17^vii^	3.3327
H14A···H17B	2.8510	H15B···C20^vii^	3.3105
H14A···H18C	2.3186	H15B···H1^vii^	2.9039
H15B···H16A	2.3082	H15B···H2^xi^	2.4244
H15B···H17B	2.4072	H15B···H11^vii^	2.7129
H15B···H18C	2.8493	H15B···H12^vii^	3.5653
Cl1···H5A^iii^	3.1443	H16A···O2^vii^	2.5834
Cl1···H6B^x^	2.9705	H16A···O4^vi^	3.0967
Cl1···H7A^iii^	3.3484	H16A···C4^xi^	3.3578
Cl1···H9C^xi^	3.0218	H16A···C5^xi^	3.5386
Cl1···H12^vii^	2.9460	H16A···C10^vii^	3.4673
Cl1···H13^i^	3.4612	H16A···H1^vii^	2.9874
Cl2···H4^v^	3.1862	H16A···H2^xi^	2.7246
Cl2···H4^ix^	3.1952	H16A···H3^xi^	3.1037
Cl2···H7A^xii^	3.2436	H16A···H10^vi^	3.5807
O1···H2^xi^	3.5966	H16A···H13^iv^	3.5989
O1···H11^iii^	2.3474	H17B···N2^iv^	3.4348
O1···H15B^xi^	2.9240	H17B···C4^xi^	3.1853
O2···H2^iii^	3.4958	H17B···C5^xi^	3.3435
O2···H3^iii^	3.5724	H17B···H2^xi^	2.4712
O2···H8B^viii^	3.1553	H17B···H3^xi^	2.8161
O2···H10^iv^	2.3840	H18C···O4^vi^	3.3897
O2···H16A^v^	2.5834	H18C···N2^iv^	2.6158
O3···H3^xi^	3.5471	H18C···C13^iv^	3.0680
O3···H11^iii^	3.3776	H18C···C19^iv^	3.3135
O4···H1^iv^	2.5687	H18C···C21^iv^	2.9127
O4···H14A^vi^	2.4859	H18C···H10^iv^	3.4202
O4···H16A^vi^	3.0967	H18C···H13^iv^	3.3482
O4···H18C^vi^	3.3897	H18C···H14A^vi^	3.0792
O5···H12^vii^	3.4785		
			
C10—O3—C11	114.2 (2)	N2—C21—C19	117.8 (2)
C22—O5—C23	116.9 (3)	N2—C21—C20	122.8 (2)
C1—N1—C9	120.29 (18)	C19—C21—C20	119.4 (3)
C1—N1—C15	118.8 (3)	O4—C22—O5	125.3 (3)
C9—N1—C15	120.4 (2)	O4—C22—C14	122.1 (3)
C13—N2—C21	117.9 (2)	O5—C22—C14	112.7 (3)
N1—C1—C2	124.4 (3)	O5—C23—C24	111.6 (3)
C1—C2—C3	119.9 (3)	N1—C1—H1	117.786
C1—C2—C10	114.2 (3)	C2—C1—H1	117.790
C3—C2—C10	125.88 (19)	C5—C4—H2	119.465
O1—C3—C2	125.3 (3)	C8—C4—H2	119.451
O1—C3—C8	120.6 (3)	C4—C5—H3	120.608
C2—C3—C8	114.06 (19)	C6—C5—H3	120.608
C5—C4—C8	121.1 (2)	C6—C7—H4	120.721
C4—C5—C6	118.8 (3)	C9—C7—H4	120.707
Cl1—C6—C5	120.1 (3)	O3—C11—H5A	110.202
Cl1—C6—C7	118.02 (17)	O3—C11—H6B	110.200
C5—C6—C7	121.9 (3)	C12—C11—H5A	110.211
C6—C7—C9	118.6 (2)	C12—C11—H6B	110.205
C3—C8—C4	118.94 (19)	H5A—C11—H6B	108.490
C3—C8—C9	122.3 (3)	C11—C12—H7A	109.471
C4—C8—C9	118.7 (3)	C11—C12—H8B	109.470
N1—C9—C7	120.18 (19)	C11—C12—H9C	109.479
N1—C9—C8	118.8 (3)	H7A—C12—H8B	109.465
C7—C9—C8	121.0 (3)	H7A—C12—H9C	109.468
O2—C10—O3	122.7 (3)	H8B—C12—H9C	109.475
O2—C10—C2	123.53 (19)	N2—C13—H10	117.798
O3—C10—C2	113.8 (3)	C14—C13—H10	117.799
O3—C11—C12	107.5 (3)	C17—C16—H11	119.933
N2—C13—C14	124.4 (3)	C20—C16—H11	119.934
C13—C14—C15	117.7 (3)	C16—C17—H12	120.148
C13—C14—C22	116.4 (3)	C18—C17—H12	120.160
C15—C14—C22	125.9 (2)	C18—C19—H13	120.507
N1—C15—C14	121.1 (2)	C21—C19—H13	120.503
N1—C15—C20	118.5 (3)	O5—C23—H14A	109.305
C14—C15—C20	120.3 (2)	O5—C23—H15B	109.313
C17—C16—C20	120.1 (3)	C24—C23—H14A	109.316
C16—C17—C18	119.7 (3)	C24—C23—H15B	109.312
Cl2—C18—C17	118.1 (3)	H14A—C23—H15B	107.959
Cl2—C18—C19	119.79 (18)	C23—C24—H16A	109.468
C17—C18—C19	122.1 (3)	C23—C24—H17B	109.468
C18—C19—C21	119.0 (2)	C23—C24—H18C	109.468
C15—C20—C16	123.6 (2)	H16A—C24—H17B	109.471
C15—C20—C21	116.9 (3)	H16A—C24—H18C	109.475
C16—C20—C21	119.5 (2)	H17B—C24—H18C	109.478
			
C10—O3—C11—C12	177.9 (2)	C3—C8—C9—N1	3.0 (4)
C10—O3—C11—H5A	57.8	C3—C8—C9—C7	−177.6 (3)
C10—O3—C11—H6B	−61.9	C4—C8—C9—N1	−178.3 (3)
C11—O3—C10—O2	−0.4 (4)	C4—C8—C9—C7	1.1 (4)
C11—O3—C10—C2	178.5 (2)	O3—C11—C12—H7A	−59.5
C22—O5—C23—C24	79.0 (3)	O3—C11—C12—H8B	−179.5
C22—O5—C23—H14A	−42.1	O3—C11—C12—H9C	60.5
C22—O5—C23—H15B	−160.0	H5A—C11—C12—H7A	60.6
C23—O5—C22—O4	−0.1 (4)	H5A—C11—C12—H8B	−59.4
C23—O5—C22—C14	177.69 (18)	H5A—C11—C12—H9C	−179.4
C1—N1—C9—C7	−179.3 (2)	H6B—C11—C12—H7A	−179.7
C1—N1—C9—C8	0.1 (4)	H6B—C11—C12—H8B	60.3
C9—N1—C1—C2	−1.5 (4)	H6B—C11—C12—H9C	−59.7
C9—N1—C1—H1	178.5	N2—C13—C14—C15	1.9 (4)
C1—N1—C15—C14	73.6 (3)	N2—C13—C14—C22	−177.6 (3)
C1—N1—C15—C20	−102.8 (3)	H10—C13—C14—C15	−178.1
C15—N1—C1—C2	−173.7 (2)	H10—C13—C14—C22	2.4
C15—N1—C1—H1	6.3	C13—C14—C15—N1	−177.0 (2)
C9—N1—C15—C14	−98.5 (3)	C13—C14—C15—C20	−0.7 (4)
C9—N1—C15—C20	85.0 (3)	C13—C14—C22—O4	28.4 (4)
C15—N1—C9—C7	−7.3 (4)	C13—C14—C22—O5	−149.5 (3)
C15—N1—C9—C8	172.1 (2)	C15—C14—C22—O4	−151.1 (3)
C13—N2—C21—C19	176.4 (3)	C15—C14—C22—O5	31.0 (4)
C13—N2—C21—C20	−1.5 (4)	C22—C14—C15—N1	2.5 (4)
C21—N2—C13—C14	−0.8 (4)	C22—C14—C15—C20	178.8 (3)
C21—N2—C13—H10	179.2	N1—C15—C20—C16	−4.0 (4)
N1—C1—C2—C3	−0.2 (4)	N1—C15—C20—C21	175.0 (2)
N1—C1—C2—C10	−179.0 (2)	C14—C15—C20—C16	179.5 (3)
H1—C1—C2—C3	179.8	C14—C15—C20—C21	−1.4 (4)
H1—C1—C2—C10	1.0	C17—C16—C20—C15	176.3 (3)
C1—C2—C3—O1	−176.1 (3)	C17—C16—C20—C21	−2.7 (4)
C1—C2—C3—C8	3.0 (4)	C20—C16—C17—C18	−0.5 (4)
C1—C2—C10—O2	6.7 (4)	C20—C16—C17—H12	179.5
C1—C2—C10—O3	−172.1 (2)	H11—C16—C17—C18	179.5
C3—C2—C10—O2	−172.0 (3)	H11—C16—C17—H12	−0.5
C3—C2—C10—O3	9.1 (4)	H11—C16—C20—C15	−3.7
C10—C2—C3—O1	2.5 (5)	H11—C16—C20—C21	177.3
C10—C2—C3—C8	−178.3 (3)	C16—C17—C18—Cl2	−176.6 (3)
O1—C3—C8—C4	−4.0 (4)	C16—C17—C18—C19	2.8 (5)
O1—C3—C8—C9	174.8 (3)	H12—C17—C18—Cl2	3.4
C2—C3—C8—C4	176.8 (2)	H12—C17—C18—C19	−177.2
C2—C3—C8—C9	−4.5 (4)	Cl2—C18—C19—C21	177.74 (17)
C5—C4—C8—C3	178.2 (3)	Cl2—C18—C19—H13	−2.3
C5—C4—C8—C9	−0.6 (4)	C17—C18—C19—C21	−1.7 (4)
C8—C4—C5—C6	−0.6 (5)	C17—C18—C19—H13	178.3
C8—C4—C5—H3	179.4	C18—C19—C21—N2	−179.7 (3)
H2—C4—C5—C6	179.4	C18—C19—C21—C20	−1.6 (4)
H2—C4—C5—H3	−0.6	H13—C19—C21—N2	0.3
H2—C4—C8—C3	−1.8	H13—C19—C21—C20	178.4
H2—C4—C8—C9	179.4	C15—C20—C21—N2	2.6 (4)
C4—C5—C6—Cl1	−179.6 (3)	C15—C20—C21—C19	−175.3 (2)
C4—C5—C6—C7	1.3 (5)	C16—C20—C21—N2	−178.2 (3)
H3—C5—C6—Cl1	0.3	C16—C20—C21—C19	3.8 (4)
H3—C5—C6—C7	−178.7	O5—C23—C24—H16A	172.0
Cl1—C6—C7—C9	−179.81 (17)	O5—C23—C24—H17B	52.0
Cl1—C6—C7—H4	0.2	O5—C23—C24—H18C	−68.0
C5—C6—C7—C9	−0.7 (4)	H14A—C23—C24—H16A	−67.0
C5—C6—C7—H4	179.3	H14A—C23—C24—H17B	173.1
C6—C7—C9—N1	178.9 (3)	H14A—C23—C24—H18C	53.0
C6—C7—C9—C8	−0.5 (4)	H15B—C23—C24—H16A	51.0
H4—C7—C9—N1	−1.1	H15B—C23—C24—H17B	−69.0
H4—C7—C9—C8	179.5	H15B—C23—C24—H18C	171.0

Symmetry codes: (i) −*x*+1, −*y*, −*z*+1; (ii) −*x*−1, −*y*, −*z*+1; (iii) −*x*+1, −*y*+1, −*z*+2; (iv) −*x*+1, −*y*+1, −*z*+1; (v) *x*−1, *y*, *z*; (vi) −*x*+2, −*y*+1, −*z*+1; (vii) *x*+1, *y*, *z*; (viii) −*x*+1, −*y*+2, −*z*+2; (ix) −*x*, −*y*, −*z*+1; (x) *x*, *y*−1, *z*; (xi) −*x*+2, −*y*+1, −*z*+2; (xii) *x*−1, *y*−1, *z*−1; (xiii) *x*+1, *y*+1, *z*+1; (xiv) *x*, *y*+1, *z*.

### Hydrogen-bond geometry (Å, º)

**Table:**

*D*—H···*A*	*D*—H	H···*A*	*D*···*A*	*D*—H···*A*
C16—H11···O1^iii^	0.95	2.35	3.231 (3)	155 (1)
C13—H10···O2^iv^	0.95	2.38	3.301 (4)	162 (1)

Symmetry codes: (iii) −*x*+1, −*y*+1, −*z*+2; (iv) −*x*+1, −*y*+1, −*z*+1.
